# Pre-treatment Pain Symptoms Influence Antidepressant Response to Ketamine in Depressive Patients

**DOI:** 10.3389/fpsyt.2022.793677

**Published:** 2022-03-16

**Authors:** Xiaofeng Lan, Yanling Zhou, Chengyu Wang, Weicheng Li, Fan Zhang, Haiyan Liu, Ling Fu, Kai Wu, Roger S. McIntyre, Yuping Ning

**Affiliations:** ^1^The Affiliated Brain Hospital of Guangzhou Medical University, Guangzhou, China; ^2^Guangdong Engineering Technology Research Center for Translational Medicine of Mental Disorders, Guangzhou, China; ^3^The First School of Clinical Medicine, Southern Medical University, Guangzhou, China; ^4^Department of Biomedical Engineering, School of Materials Science and Engineering, South China University of Technology, Guangzhou, China; ^5^Canadian Rapid Treatment Center of Excellence, Mississauga, ON, Canada; ^6^Mood Disorders Psychopharmacology Unit, Poul Hansen Family Centre for Depression, University Health Network, Toronto, ON, Canada; ^7^Department of Psychiatry, University of Toronto, Toronto, ON, Canada; ^8^Institute of Medical Science, University of Toronto, Toronto, ON, Canada; ^9^Brain and Cognition Discovery Foundation, Toronto, ON, Canada; ^10^Department of Pharmacology and Toxicology, University of Toronto, Toronto, ON, Canada

**Keywords:** ketamine, depression, pain, bipolar depression, treatment resistant, moderate

## Abstract

**Background:**

Pain strongly coexists with depression. Ketamine has great analgesic and antidepressant effects, acting as a promising role in treating depression with pain. Few studies have evaluated impact of pain symptoms on antidepressant effect of ketamine infusions. Thus, present study investigated whether pain symptoms in individuals with depression moderate response to ketamine.

**Methods:**

One hundred and four individuals with major depressive disorder and bipolar depression received six intravenous infusions of ketamine. The Montgomery–Åsberg Depression Rating Scale (MADRS) was administered at baseline, the next morning after each infusion and 2 weeks (Day 26) after the last infusion. Pain symptoms were collected at baseline using the short-form McGill Pain Questionnaire (SF-MPQ).

**Results:**

The prevalence of pain in patients with depression was 48.8%. Mix model analyses showed that pre-treatment pain symptoms assessed by each domain of SF-MPQ significantly moderated antidepressant response to six infusions of ketamine from baseline to day 26 (all *p* < 0.05). Then follow-up simple slopes analyses suggested that all patients across groups showed a significant symptomatic improvement after ketamine infusions (all *p* < 0.05), and patients with severe pain (across all domains of SF-MPQ) had greater improvement in depressive symptoms than those with mild pain or non-pain (all *p* < 0.05).

**Conclusion:**

A significant and rapid improvement in depressive symptoms was observed in patients with depression and pain after ketamine treatment. Ketamine may be a novel and promising antidepressant preferentially for the therapy of depression with severe pain.

## Introduction

Both depression and pain are widespread and debilitating conditions, contributing to substantial disability as well as large socioeconomic burden worldwide (1, 2). The foregoing conditions are reported to be strongly comorbid in clinical settings (3). For example, it has been reported that the prevalence of pain among individuals affected by depression ranges from 43.3 to 80% (4–6). Moreover, comorbidity of pain and depression is reported to result in greater function impairment, increased health care costs and decreased quality of life (7–9) when compared to the effect of either condition occurring alone in affected individuals. Additionally, symptoms of pain have been associated with a greater treatment resistance in persons with depression (10, 11). Evidence from the Sequenced Treatments for Alternatives to Relieve Depression (STAR*D) study demonstrates that baseline pain severity is associated with more severe depressive symptoms and poorer treatment response to conventional antidepressants in individuals with major depressive disorder (MDD) (5). Improving treatment outcomes in individuals affected by depression with pain symptoms becomes the top therapeutic priority from an individual and socio-economic perspective.

In recent decades, intensified efforts are underway to develop new antidepressants. Ketamine is a non-selective N-methyl-D-aspartic acid receptor (NMDAR) antagonist with anesthetic and analgesic effects, which can effectively relieve acute and chronic pain (12–15). Ketamine has also been reported to have rapid antidepressant and anti-suicidal effects in patients with MDD or bipolar depression (BD) in recent years (16–18). The foregoing highly replicated finding is observed in both open-label studies and randomized, placebo-controlled trials (18–20). Although a single infusion of ketamine at a subanesthetic dose (0.5 mg/kg) can quickly improve depressive symptoms, its antidepressant effect only lasts a few days (19). Repeat-dose infusions of ketamine can extend the antidepressant effect and achieve treatment response for weeks (18, 21, 22). Moreover, our previous publication reported that only 14% of patients with MDD and BD achieved response after the first ketamine infusion while up to 68% of patients responded to six infusions of ketamine (23).

Ketamine has also been reported to be effective in individuals with the comorbidity of depression pain (24, 25). A recent study reported that oral therapy of ketamine for 6 weeks can successfully alleviate depressive symptoms in chronic pain patients (26). Moreover, our recent publication reported that subanesthetic infusions of ketamine can both reduce depressive symptoms and pain symptoms in individuals with treatment resistant depression (TRD) (27).

Taken together, ketamine may be a promising treatment for individuals experiencing comorbid depression and pain. However, there is limited clinical research that has evaluated the role of pre-treatment pain symptoms in response to repeated ketamine infusions in individuals with depression. Thus, the study herein aimed to determine whether pre-treatment pain symptoms moderate the antidepressant efficacy of repeated intravenous ketamine infusions in individuals with MDD or BD. Considering the powerful effect of ketamine on alleviating pain, we hypothesize that pre-treatment pain severity may differently moderate the response to ketamine infusions in patients with depression when compared to conventional antidepressant treatment.

## Materials and Methods

Present study was a single-arm open-label clinical trial of six ketamine infusions conducted in The Affiliated Brain Hospital of Guangzhou Medical University from November 2016 to July 2018 which aimed to explore the antidepressant efficacy of adjunctive ketamine in patients with MDD or BD. The protocol was approved by the Clinical Research Ethics Committee of The Affiliated Brain Hospital of Guangzhou Medical University with a clinical trial number, ChiCTROOC-17012239. All participants provided informed consent before entering our study. Patient selection and study design are introduced briefly in our current study, as they have been described in detail in our previous study (23).

### Participants

Participants who satisfied the following inclusion criteria were permitted to participate in our study: (1) aged 18∼65 years old; (2) were suffering from MDD or BD without psychotic symptoms according to the structured clinical interview for Diagnostic and Statistical Manual of Mental Disorders, fifth edition (DSM-5); (3) were experiencing moderate to severe depressive symptoms defined as a score of ≥17 on the 17-item Hamilton Depression Rating scale (HAMD-17) (28), and were suffering from current suicidal ideation with a score of ≥2 on Beck Scale for Suicide Ideation-part I (29) or had a history of TRD who did not respond to two or more classes of antidepressants with adequate dosage and treatment duration (30). The exclusion criteria included (1) substance use disorder; (2) any serious or unstable physical disease or neurological illness; (3) any other severe mental disorders (i.e., dementing disorders or schizophrenia); (4) pregnant or breast-feeding. In addition, patients’ oral psychiatric medications were required to be administered at a stable dose for more than 4 weeks before the trial and maintained throughout the infusion period.

### Study Design

Participants received six intravenous infusions of ketamine for 2 weeks (three times per week, Monday-Wednesday-Friday). After an overnight fast, enrolled patients received an intravenous (IV) infusion of ketamine dosed at 0.5 mg/kg diluted in 0.9% saline, which was delivered at least 40 min. IV ketamine infusions were performed on days 1, 3, 5, 8, 10, and 12 during the trial.

### Rating Scales

Depressive symptoms were measured by the Montgomery–Åsberg Depression Rating Scale (MADRS) (31), which was administered at baseline, the next morning (24 h) after each infusion, and 2 weeks (Day 26) after the last infusion. Pain severity was assessed by the short-form McGill Pain Questionnaire (SF-MPQ) (32). The SF-MPQ was used to assess pain severity with sufficient reliability and validity, which had three sections: (1) A visual analog scale (VAS) was used to evaluate the intensity of subjective pain with scores from 0 (non-pain) to 10 (worst imaginable pain). (2) The pain rating index (PRI) consisted of sensory index and affective index, providing with a total of 15 items scored from 0 (non-pain) to 3 (severe pain). (3) Present pain intensity (PPI) was used to measure current pain intensity from 0 (no pain) to 5 (excruciating).

### Statistical Analyses

The retrospective exploratory analysis was conducted in SPSS 22.0 statistical software. All tests were two-sided with significance at *p* < 0.05. Student’s *t*-test for continuous variables and the chi-square test categorical variables were used to compare demographic variables and clinical data between the groups (pain vs. non-pain). Analysis of variance was performed to compare differences in pre-treatment depressive severity between severe pain, mild pain, and non-pain groups. Bonferroni test was used in *post hoc* comparisons. With respect to missing data, a mixed model was performed to assess change in MADRS scores from baseline to day 26. The interaction effect between ketamine treatment and pre-treatment pain severity was calculated to evaluate whether overall changes in depressive symptoms measured by MADRS score from baseline to day 26, were moderated by pre-treatment pain symptoms, as measured by VAS, sensory index, affective index, PPI and their total score. Follow-up simple slopes comparisons were performed for significant interactions, comparing difference of slopes between mild, severe pain and non-pain groups. Patients exhibiting no pain symptoms at pre-treatment were characterized as “non-pain,” patients exhibiting the mean or below the average score (i.e., VAS, sensory index, affective index, PPI, or total pain) were characterized as “mild pain” and patients exhibiting above the average score were characterized as “severe pain.” Therefore, five separate models were conducted in our statistical analyses. Age, gender, body mass index (BMI), education, duration of illness, diagnosis, and pre-treatment depression severity were included as covariates in each model. In addition, pre-treatment MADRS score was evaluated for multicollinearity with pre-treatment pain symptoms before entering the model.

## Results

A total of 104 depressive patients with pre-treatment pain data received six infusions of ketamine. 16 patients dropped out at day 26. Thus, depressive symptoms assessed by MADRS were available for 104 patients at pre-treatment and for 88 patients at day 26. 48.8% of patients (*N* = 50) were suffering pain before ketamine treatment. Baseline sociodemographic and clinical characteristics are presented in Table 1.

**TABLE 1:** A summary of depressive patient’s demographic and clinical characteristics.

Variables	Non-pain (*N* = 54)		Pain (*N* = 50)		X^2^	*P*
	*N*	%	*N*	%		
Gender (male)	26	48.1	26	52.0	0.154	0.845
Employment status (working)	27	50.0	18	36.0	2.073	0.150
Smoking	9	16.7	9	18.0	0.032	0.857
TRD	45	83.3	44	88.0	0.458	0.499
With suicidality	44	81.5	35	70.0	1.874	0.171
Psychiatric comorbidity (yes)^①^	13	24.1	7	14.0	1.696	0.193
Having family history of psychiatric disorders	18	33.3	24	48.0	2.320	0.128
Previous hospitalization (yes)^②^	17	31.5	14	28.0	0.150	0.698
Current pharmacotherapies						
≥2 antidepressant	8	14.8	10	20.0	0.488	0.485
Mood stabilizer	16	29.6	14	28.0	0.034	0.855
Benzodiazepine	28	51.9	22	44.0	0.641	0.423
Antipsychotic	31	57.4	24	48.0	0.922	0.337
	**Mean**	**SD**	**Mean**	**SD**	***t***	***P***
Age (years)	32.7	10.9	36.0	12.1	–1.450	0.150
Education (years)	13.1	2.9	11.4	3.3	2.761	0.007
Duration of illness (months)	95.0	76.0	116.9	104.7	–1.226	0.223
BMI (kg/m^2^)	22.2	3.6	23.4	3.5	–1.692	0.094
Dose of antidepressant (mg/day)^③^	36.6	23.4	39.0	22.0	–0.525	0.601
Pre-treatment MADRS score	31.5	8.2	32.9	6.7	–1.206	0.231
Pre-treatment VAS score	−	−	5.2	2.0	NA	NA
Pre-treatment sensory index	−	−	4.1	3.1	NA	NA
Pre-treatment affective index	−	−	4.7	2.9	NA	NA
Pre-treatment present pain intensity	−	−	2.4	1.0	NA	NA
Pre-treatment total pain	−	−	16.5	7.2	NA	NA

*MDD, Major Depressive Disorder; TRD, Treatment-resistant Depression; BMI, body mass index; MADRS, Montgomery-Åsberg Depression Rating Scale; VAS, visual analog scale; NA, not applicable. ①Comorbidity of an Axis I anxiety disorder, obsessive-compulsive disorder, phobia, or panic disorder. ②Previous hospitalization due to mental health problems. ③Fluoxetine equivalent dose.*

According to mean scores of VAS, sensory index, affective index, PPI, and total pain, there were 23, 32, 24, 26, and 24 patients with mild pain, respectively, as well as 27, 18, 26, 24, and 26 patients with severe pain, respectively. As shown in Table 2, pre-treatment MADRS scores were significantly different among non-pain, mild pain and severe pain groups. *Post hoc* comparisons using the Bonferroni test indicated that the mean pre-treatment MADRS score of patients with severe sensory index (mean = 37.6 ± 5.1) was significantly higher than patients with mild sensory index (mean = 30.5 ± 6.6, *p* = 0.008) or patients without pain (mean = 31.5 ± 8.2, *p* = 0.004). However, the mean pre-treatment MADRS score did not significantly differ between patients with mild sensory index and patients without pain (*p* = 1.000). Similar findings were observed when comparation were performed among patients with mild/severe affective index and non-pain, with mild/severe PPI and non-pain, as well as mild/severe total pain and non-pain, consistently suggesting that pre-treatment MADRS score in patients with severe pain was higher than patients with mild pain or non-pain (all *p* < 0.05). Mean pre-treatment MADRS scores in patients with severe VAS (mean = 35.3 ± 6.9) was also higher than patients with mild VAS (mean = 30.4 ± 6.3), but this difference didn’t reach a statistical significance (*p* = 0.093). Similarly, mean pre-treatment MADRS scores in patients with severe VAS was higher than patients without pain (mean = 31.5 ± 8.2), but this difference did not reach a statistical significance either (*p* = 0.068).

**TABLE 2:** Comparisons of baseline depressive severity among severe, mild, and non-pain group.

Variable	Non-pain	Mild pain	Severe pain	*F*	*P^a^*
**Visual analog scale**	*N* = 54	*N* = 23	*N* = 27		
Pre-treatment MADRS score	31.5 ± 8.2	30.4 ± 6.3	35.3 ± 6.9	3.271	0.042^b^
**Sensory index**	*N* = 54	*N* = 32	*N* = 18		
Pre-treatment MADRS score	31.5 ± 8.2	30.5 ± 6.6	37.6 ± 5.1	6.082	0.003^c^
**Affective index**	*N* = 54	*N* = 24	*N* = 26		
Pre-treatment MADRS score	31.5 ± 8.2	29.8 ± 7.2	36.1 ± 5.3	5.264	0.007^c^
**Present pain intensity**	*N* = 54	*N* = 26	*N* = 24		
Pre-treatment MADRS score	31.5 ± 8.2	29.8 ± 5.4	36.6 ± 6.9	6.109	0.003^c^
**Total pain**	*N* = 54	*N* = 24	*N* = 26		
Pre-treatment MADRS score	31.5 ± 8.2	28.8 ± 5.8	37.0 ± 5.5	9.107	<0.001^c^

*MADRS, Montgomery-Åsberg Depression Rating Scale.*

*^a^p-value represents overall F-test. Post hoc comparisons listed as significant p < 0.05 using Bonferroni test.*

*^b^None of Bonferroni tests are statistically significant.*

*^c^Comparisons of severe vs. mild and non-pain are significant.*

**FIGURE 1:**
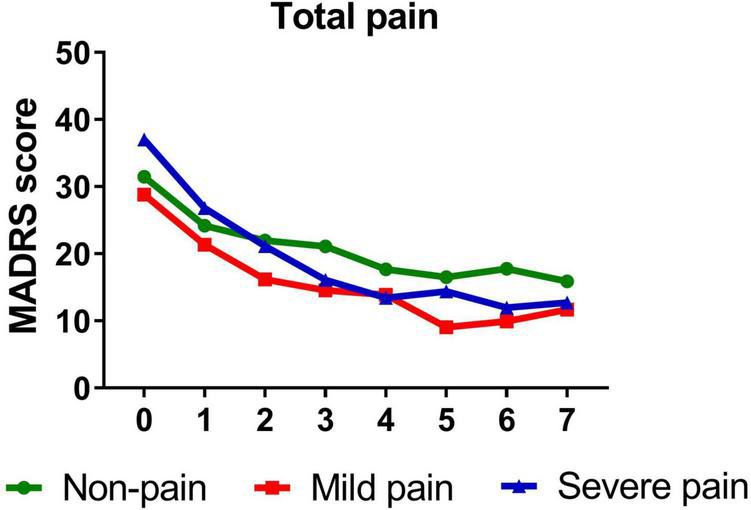
Overall change in MADRS score across six infusions. Pain severity was defined by total score of SF-MPQ. Patients exhibiting no pain symptom were characterized as “non-pain,” patients exhibiting the mean or below the mean total pain score were characterized as “mild pain” and patients exhibiting above the mean total pain score were characterized as “severe pain.” MADRS, Montgomery-Åsberg Depression Rating Scale; SF-MPQ, short-form McGill Pain Questionnaire. Numbers on *X*-axis: 0 represents baseline, 1∼6 represent the next morning after each infusion, 7 represents 2 weeks after infusions (Day 26).

Table 3 presents standardized coefficients, standard errors and *P*-value for each model. In summary, total pain significantly moderated changes in depressive symptoms from baseline to day 26 (*B* = −0.344, *t* = −5.214, *P* < 0.001). Follow-up simple slopes analyses showed that patients with mild total pain (*B* = −1.246, *t* = −12.051, *P* < 0.001), severe total pain (*B* = −1.706, *t* = −13.684, *P* < 0.001), and non-pain (*B* = −1.004, *t* = −12.976, *P* < 0.001) all reported significant reductions in depressive symptoms from baseline to day 26. Figure 1 shows that patients with severe pain obtained a more robust symptomatic improvement than mild pain or non-pain patients (slope: severe pain > mild pain, non-pain).

**TABLE 3:** Results from mixed models for the moderating effects of pain symptom on changes in overall depressive symptoms from pre-treatment to post-infusion.

Parameter	*B*	SE	*t*	*P*	95% C.I. for B	
					Lower	Upper
**Visual analog scale**						
Ketamine	–0.992	0.072	–13.700	<0.001	–1.134	–0.850
Pre-treatment pain	2.659	0.653	4.074	<0.001	1.378	3.940
Ketamine × Pre-treatment pain	–0.332	0.065	–5.089	<0.001	–0.461	–0.204
**Sensory index**						
Ketamine	–1.017	0.072	–14.065	<0.001	–1.159	–0.875
Pre-treatment pain	2.836	0.724	3.916	<0.001	1.415	4.258
Ketamine × Pre-treatment pain	–0.336	0.073	–4.601	<0.001	–0.479	–0.192
**Affective index**						
Ketamine	–1.021	0.073	–14.040	<0.001	–1.164	–0.878
Pre-treatment pain	2.503	0.664	3.768	<0.001	1.199	3.807
Ketamine × Pre-treatment pain	–0.294	0.066	–4.446	<0.001	–0.423	–0.164
**Present pain intensity**						
Ketamine	–0.989	0.072	–13.678	<0.001	–1.131	–0.847
Pre-treatment pain	2.815	0.669	4.206	<0.001	1.501	4.129
Ketamine × Pre-treatment pain	–0.346	0.067	–5.166	<0.001	–0.477	–0.214
**Total pain**						
Ketamine	–0.986	0.072	–13.633	<0.001	–1.128	–0.844
Pre-treatment pain	2.933	0.657	4.462	<0.001	1.643	4.224
Ketamine × Pre-treatment pain	–0.344	0.066	–5.214	<0.001	–0.474	–0.215
						

**FIGURE 2:**
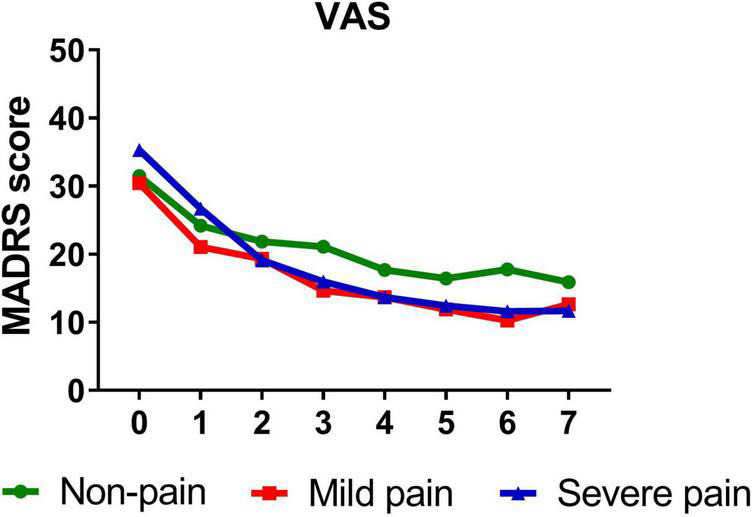
Overall change in MADRS score across six infusions. Pain severity was defined by VAS. Patients exhibiting no pain symptom were characterized as “non-pain,” patients exhibiting the mean or below the mean VAS score were characterized as “mild pain” and patients exhibiting above the mean VAS score were characterized as “severe pain.” MADRS, Montgomery-Åsberg Depression Rating Scale; VAS, visual analog scale. Numbers on *X*-axis: 0 represents baseline, 1∼6 represent the next morning after each infusion, 7 represents 2 weeks after infusions (Day 26).

A similar pattern was observed when VAS (*B* = −0.332, *t* = −5.089, *P* < 0.001), sensory index (*B* = −0.336, *t* = −4.601, *P* < 0.001), affective index (*B* = −0.294, *t* = −4.446, *P* < 0.001), and PPI (*B* = −0.346, *t* = −5.166, *P* < 0.001) were, respectively, included as moderators, such that all significantly moderated antidepressant effect of IV ketamine treatment. Participants with mild VAS (*B* = −1.266, *t* = −11.070, *P* < 0.001) and severe VAS (*B* = −1.675, *t* = −14.629, *P* < 0.001) both reported significant reductions in depressive symptoms from baseline to day 26. Figure 2 shows that patients with severe pain obtained a more robust symptomatic improvement than mild pain or non-pain patients (slope: severe pain > mild pain, non-pain).

**FIGURE 3:**
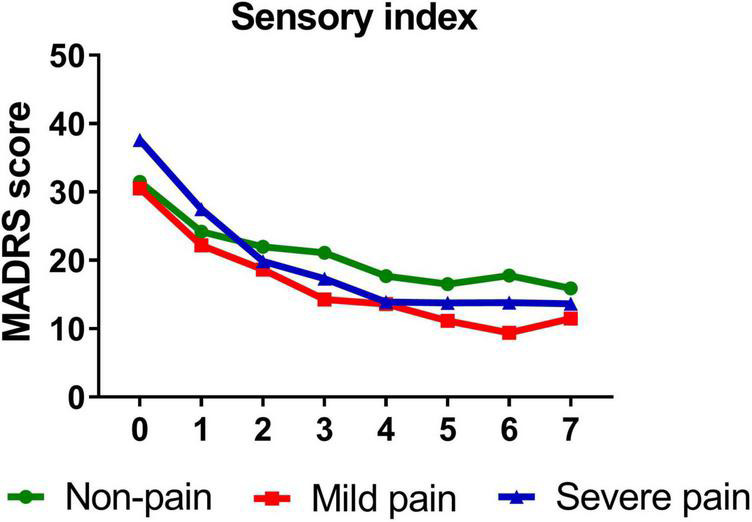
Overall change in MADRS score across six infusions. Pain severity was defined by sensory index. Patients exhibiting no pain symptom were characterized as “non-pain,” patients exhibiting the mean or below the mean sensory index were characterized as “mild pain” and patients exhibiting above the mean sensory index were characterized as “severe pain.” MADRS, Montgomery-Åsberg Depression Rating Scale. Numbers on *X*-axis: 0 represents baseline, 1∼6 represent the next morning after each infusion, 7 represents 2 weeks after infusions (Day 26).

**FIGURE 4:**
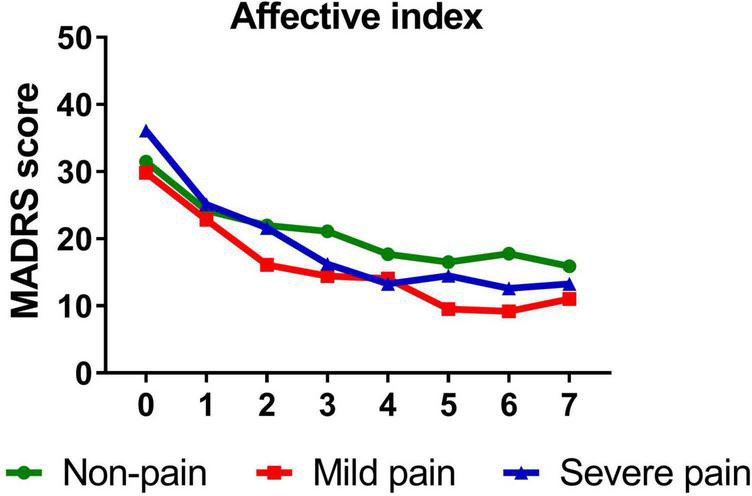
Overall change in MADRS score across six infusions. Pain severity was defined by affective index. Patients exhibiting no pain symptom were characterized as “non-pain,” patients exhibiting the mean or below the mean affective index were characterized as “mild pain” and patients exhibiting above the mean affective index were characterized as “severe pain.” MADRS, Montgomery-Åsberg Depression Rating Scale. Numbers on *X*-axis: 0 represents baseline, 1∼6 represent the next morning after each infusion, 7 represents 2 weeks after infusions (Day 26).

**FIGURE 5:**
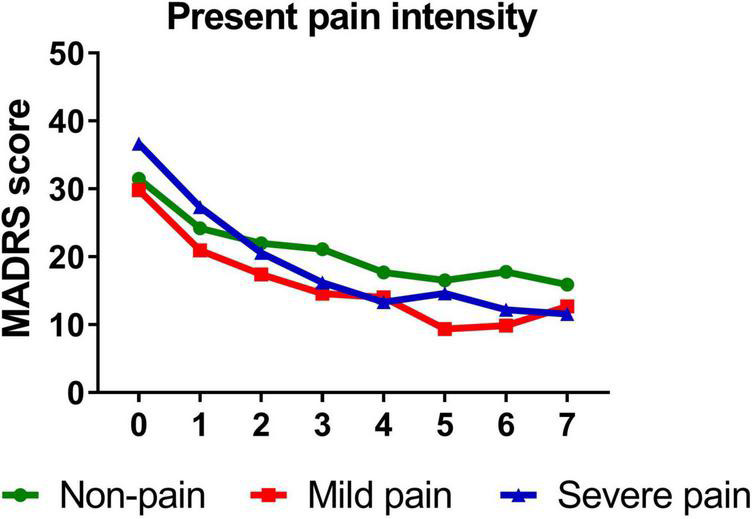
Overall change in MADRS score across six infusions. Pain severity was defined by present pain intensity. Patients exhibiting no pain symptom were characterized as “non-pain,” patients exhibiting the mean or below the mean present pain intensity were characterized as “mild pain” and patients exhibiting above the mean present pain intensity were characterized as “severe pain.” MADRS, Montgomery-Åsberg Depression Rating Scale. Numbers on *X*-axis: 0 represents baseline, 1∼6 represent the next morning after each infusion, 7 represents 2 weeks after infusions (Day 26).

Similarly, participants with mild (*B* = −1.389, *t* = −14.811, *P* < 0.001)/severe (*B* = −1.642, *t* = −10.612, *P* < 0.001) sensory index, with mild (*B* = −1.386, *t* = −12.281, *P* < 0.001)/severe (*B* = −1.568, *t* = −13.334, *P* < 0.001) affective index, or with mild (*B* = −1.263, *t* = −11.703, *P* < 0.001)/severe (*B* = −1.711, *t* = −14.048, *P* < 0.001) PPI all reported significant reductions in depressive symptoms from baseline to day 26. Figures 3–5 show that patients with mild or severe pain obtained a more robust symptomatic improvement than non-pain patients (slope: severe pain, mild pain > non-pain).

## Discussion

To our best knowledge, this is the first study to explore the impact of pre-treatment pain symptoms on antidepressant effect of repeated intravenous infusions of ketamine in individuals with depression. The main findings are: (1) the rate of pain in depressive patients was high (48.8%); (2) patients with greater pain severity also had higher pre-treatment depressive scores assessed by MADRS; (3) the benefits of repeated infusions of ketamine were observed in patients affected by MDD/BD, both with and without pain; and (4) patients affected by MDD/BD with higher pre-treatment pain exhibited more robust symptomatic improvement, suggesting pre-treatment pain severity moderated ketamine’s antidepressant effect, which is inconsistent with outcomes observed with conventional antidepressants.

Nearly a half of patients with depressive symptoms in our study reported pain symptoms, which was consistent with previous reports (6, 27). This finding suggests a high proportion of patients may be resistant to antidepressant treatment, as pain symptoms could negatively impact insight of depression and adherence to medication, which may lead to adverse results (3). In accordance with previous studies (3, 5, 33), our findings suggest that patients with greater pain severity suffered from more severe depression. Increased pain severity was associated with more pain-related functional limitations (34), which may interfere with daily activities and consequently affect depression severity (3).

Our study revealed that significant and rapid reductions in depressive symptoms were observed across groups after receiving six infusions of IV ketamine, suggesting that ketamine has a robust antidepressant effect which has been supported by a wealth of studies (35–38). However, the moderational effect of pain symptoms on observed antidepressant effect of ketamine treatment in persons with depression requires further investigation.

It has been reported that pain adversely affects the treatment response to conventional antidepressants in persons with depression. For example, results from the STAR*D study indicated that MDD patients with physical pain symptoms were less likely to remit and took longer to achieve remission when they were treated by selective serotonin reuptake inhibitor (SSRI) antidepressants (5). A previous randomized controlled trial also showed that baseline pain symptoms reduced the antidepressant benefits after 3 months of treatment, and an increase in levels of baseline pain severity was associated with a decrease in the likelihood of improvement of depressive symptoms (3).

Ketamine, a novel, rapid-acting antidepressant, has been reported to have therapeutic effects in both pain and depressive symptoms, and may be an ideal treatment option for individuals with depression and comorbid pain symptoms (26, 27). For example, a double randomized controlled study showed that daily oral ketamine for 6 weeks is more effective in improving depressive symptoms in chronic pain patients comorbid with mild to moderate depression when compared to diclofenac (26). A separate study conducted by our group has shown that persons with TRD and comorbid pain symptoms are more likely to achieve response and remission than those without pain after receiving six ketamine infusions (27). The study herein extends the foregoing finding, with evidence of ketamine successfully reducing depressive symptoms in both patients with and without pain. Moreover, the study herein found that higher level of pre-treatment pain severity was associated with greater antidepressant effect of ketamine in patients with depression, which is discordant with outcomes observed with conventional antidepressants. The foregoing findings suggest that the antidepressant effect of ketamine may arise from a different neural mechanism from conventional monoamine-based antidepressants.

There may be neurobiological mechanisms underlying the enhanced ketamine’s antidepressant effect in patients with depression and severe pain. Excessive activation of inflammation has been implicated in the pathogenic mechanism of the comorbidity of depression and pain. In animal models of depression with concomitant neuropathic pain, the increased expression of pro-inflammatory cytokines is observed in brain regions that are responsible for processing emotion and pain (39, 40). Similar findings also are reported by clinical research. For example, microglial activation has been found in brain regions related to pain and emotion including the prefrontal cortex and hippocampus in patients who exhibited pain and depression (41). In addition, peripheral increased cytokines such as tumor necrosis factor (TNF)-α and interleukin (IL)-6 have been reported in individuals with the comorbidity of depression and pain (41). Our previous publication revealed that depressive patients with pain had higher plasma levels of IL-6 and granulocyte-macrophage colony stimulating factor (GM-CSF) than those without pain, suggesting pain increases inflammatory response in patients affected by depression (27).

Ketamine has anti-inflammatory effects, which may be implicated in its therapeutic effect on pain and depressive symptoms (42). Moreover, ketamine’s analgesic effect can also enhance its anti-depressive effect via alleviating immune response in depressive patients with pain (27). Therefore, inflammation may by a contributing factor to the observed moderational effect of pain on depressive symptom reduction in individuals with MD/BD.

Regarding to the adverse effects, ketamine IV three times a week in our study showed well-tolerated and safe during the infusions as the psychotomimetic and dissociative symptoms, and blood pressure and pulse elevations were very mild and transient, which have been described in detail in our previous publication (43). In recent years, most studies have conducted repeated ketamine infusions (mainly two or three times weekly) to prolong antidepressant effects. Increased infusions of ketamine may increase adverse effects. For example, it is reported that six infusions of ketamine had greater side effects as compared to single ketamine (44). But few studies have compared the side effects of twice- and thrice-weekly ketamine treatment schedules directly. Only one study has evaluated the efficacy and safety of twice- and thrice-weekly intravenous infusion of ketamine, indicating that both treatment schedules achieved similar antidepressant effect but there was no significant difference in adverse events between the two groups due to small sample size (45). Despite of that, both twice- and thrice-weekly intravenous infusion of ketamine showed acceptable short-term side effects such as psychotomimetic and dissociative symptoms, and other general adverse events (headache, nausea, dizziness, etc.) (18, 46). In addition, a longer-term follow-up study (range, 8 months to 6 years) that carried an open-label ketamine infusion three times weekly over a 12-day period showed that no persistent physical symptoms or increased substance use were found in participants, suggesting longer-term safety of ketamine treatment (46). However, considering few studies, including current study, have measured the abuse potential of ketamine, substantially more follow-up studies on the long-term safety are needed.

Several limitations should be noted. First, this was a retrospective analysis of data from an open-label clinical study without placebo controls. And the potential subjective bias of patients and researchers may affect the interpretation of the results because of the lack of blind method. Second, the sample size of this study is relatively small, which may lead to sampling bias. Moreover, due to the limited sample size, patients with either MDD or BD analyzed as a whole in this study may increase heterogeneity. Third, we only recruited depressive patients with treatment-resistant and with suicidality, which may limit the generalizability of these results for depression patients. Fourth, baseline demographic characteristics of patients with mild, severe and non-pain were not shown because they were divided into multiple groups according to different domains of SF-MPQ. Fifth, we did not measure biological indicators such as plasma cytokines which may enhance persuasive evidence. Thus, it is necessary for further study to measure biological indicators in these depressive patients.

In summary, our study showed that depressive patients with varying degrees of pain exhibited a significant and rapid improvement in depressive symptoms after six infusions of ketamine treatment, and pre-treatment pain symptoms moderated ketamine’s antidepressant effect. Overall, our findings suggest that ketamine may be a novel and promising antidepressant preferentially for the therapy of depression with severe pain.

## Data Availability Statement

The raw data supporting the conclusions of this article will be made available by the authors, without undue reservation.

## Ethics Statement

The studies involving human participants were reviewed and approved by the Clinical Research Ethics Committee of The Affiliated Brain Hospital of Guangzhou Medical University. The patients/participants provided their written informed consent to participate in this study.

## Author Contributions

YN and YZ designed the study and wrote the protocol. YN provided research supervision. XL and YZ wrote the manuscript. RM helped to revise the manuscript. All authors participated in the data collection and contributed and approved the final manuscript.

## Conflict of Interest

RM has received research grant support from CIHR/GACD/Chinese National Natural Research Foundation; and speaker/consultation fees from Lundbeck, Janssen, Purdue, Pfizer, Otsuka, Takeda, Neurocrine, Sunovion, Bausch Health, Novo Nordisk, Kris, Sanofi, Eisai, Intra-Cellular, NewBridge Pharmaceuticals, and AbbVie. RM is a CEO of Braxia Scientific Corp. The remaining authors declare that the research was conducted in the absence of any commercial or financial relationships that could be construed as a potential conflict of interest.

## Publisher’s Note

All claims expressed in this article are solely those of the authors and do not necessarily represent those of their affiliated organizations, or those of the publisher, the editors and the reviewers. Any product that may be evaluated in this article, or claim that may be made by its manufacturer, is not guaranteed or endorsed by the publisher.
